# The Value of Combined Large Format Histopathology Technique to Assess the Surgically Removed Breast Tissue following Neoadjuvant Chemotherapy: A Single Institution Study of 40 Cases

**DOI:** 10.1155/2012/361707

**Published:** 2012-10-18

**Authors:** Julio A. Ibarra

**Affiliations:** Department of Pathology, University of California and Irvine and MemorialCare Breast Center, Orange Coast Memorial Medical Center, 9920 Talbert Avenue, Fountain Valley, CA 92708, USA

## Abstract

Historically, neoadjuvant chemotherapy has been used to treat patients with advanced breast disease in an attempt to convert them into candidates for breast conservation surgery. The ultimate goal of histopathologic examination of the specimens removed after neoadjuvant chemotherapy is the identification of either residual disease or positive identification of the tumor bed. We report a series of 40 patients treated with neoadjuvant chemotherapy and evaluation of the surgical specimens by a combination of standard histopathology and the use of large format histopathology techniques.

## 1. Introduction

The use of preoperative systemic therapy has increased in the last several years. Originally this therapy was used predominantly for patients with locally advanced breast cancer without systemic disease; the purpose was to convert these inoperable patients into candidates for breast conservation surgery [1–3]. However, neoadjuvant chemotherapy has also been extended to patients without locally advanced breast cancer that traditionally were subjected to surgery as the primary treatment modality [4–7]. The definition of pathologic complete response (pCR) was proposed in the NSABP B18 and B27 protocols; it is defined as the complete absence of invasive carcinoma both in the breast and in the axillary lymph nodes. The presence of residual duct carcinoma in situ (DCIS) was acceptable for the definition of pCR in these original studies. This definition has been challenged by others, some of which include small areas of residual tumor [8] or noninvasive disease in the pathologic complete response group [9].

Regardless of the definition used, the role of the pathologist in the evaluation of the resected specimens, whether it is a mastectomy or a partial mastectomy, is the identification of residual viable tumor or documenting the presence of the tumor bed and the absence of residual tumor in cases with pathologic complete response. In order to accomplish this task, the pathologist has to work in close cooperation with the radiologist in order to determine whether there was a residual “mass” or the fiducial clip placed by the radiologist before the start of therapy. A comprehensive review on the evaluation of pathology specimens after neoadjuvant therapy was published by Sneige and Page [10]. They indicate the importance of radiology and the fact that “extensive” sampling is required for complete pathologic evaluation. There are, however, no strict guidelines regarding the volume of tissue recommended for investigation as long as the tumor bed or residual tumor are found.

## 2. Materials and Methods

The standard processing of the tissue is done by obtaining and processing blocks that measure no more than 2.5 × 2 cm from the areas of most interest according to the macroscopic (gross) evaluation by the pathologist. This works relatively well when there is a ‘‘mass” or an abnormality that is either visible or palpable by the pathologist. Many cases of neoadjuvant chemotherapy, however, will not have these changes and therefore it becomes extremely difficult to determine where to sample a breast specimen without the aid of the radiologist.

**Figure 1:**
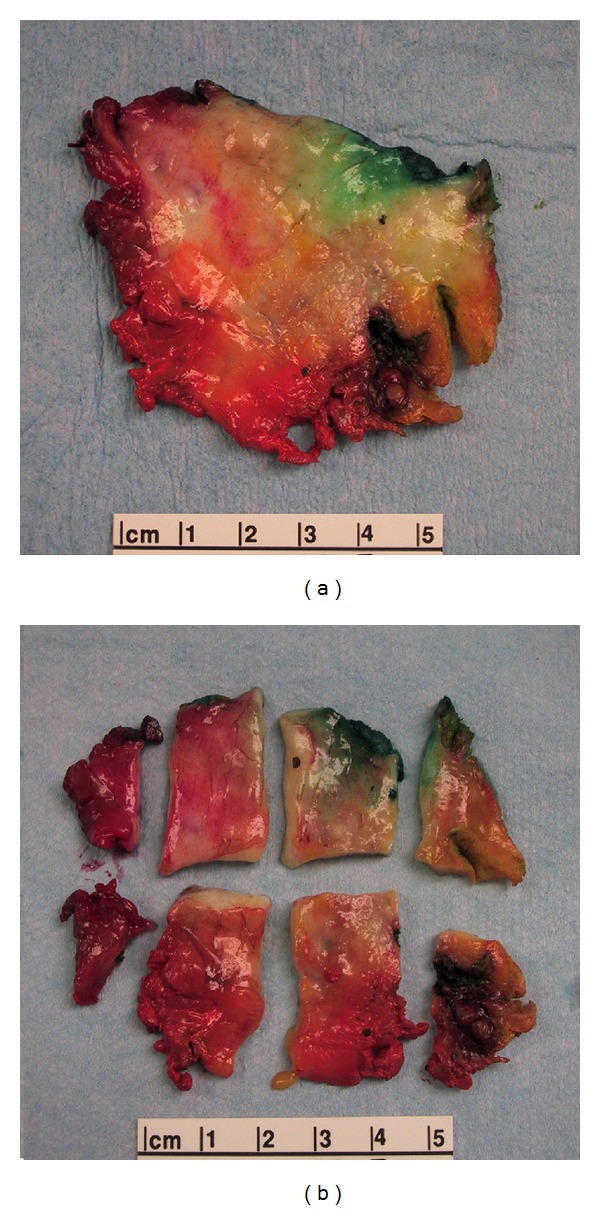
Each tissue slice will generate anywhere between 6 and 10 standard sections depending on the size. In this example of a slice measuring 6 cm in the largest dimension we created 8 generous standard sections.

**Figure 2:**
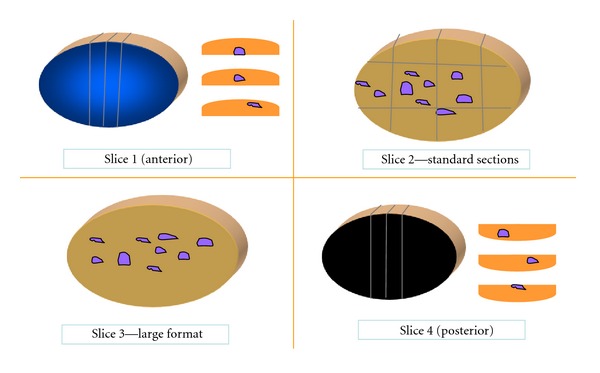
Partial mastectomy cut into four slices. Slice one (top) is anterior and slice 4 (bottom) is posterior. These two are sectioned in the perpendicular plane to evaluate those two margins microscopically. Slice 2 is cut into small pieces and entirely submitted for standard histologic sections. Slice 3 is processed intact for large format histopathology.

The tissue is inked with 6 colors for partial mastectomies and with 3 colors for mastectomies; the tissue is then sliced at 5–10 mm thick intervals with a sharp knife and the slices placed on separate paper towels. The radiology information is used to localize the residual tumor or clip that was placed preoperatively. This is accomplished with X-rays of the intact resected specimen and/or X-rays of the slices of tissue. Once the area (tumor bed) is located, it is extensively sampled. The slices that are determined to contain the residual tumor or the tumor bed are used one for large format and one for standard sections. We obtain an average of 1 large format histopathology slide per case that can measure up to 7 × 9 cm and process it with the techniques described elsewhere [11–14]. Then we submit the mirror image for standard sections; usually 8–10 standard sections are equivalent to one large format slide (Figure 1). For partial mastectomy cases, the large format and the mirror image standard sections allow us to evaluate 100% of four out of six radial margins. The other two surgical margins (top and bottom slices) are sampled by cutting perpendicular sections of those slices (Figure 2). For mastectomies we have submitted one to three large format sections and several standard sections from the tissue adjacent to the large format (average 16 per case compared to 10 in partial mastectomies); the margins are sampled as needed depending on the location of the “tumor bed” and the radiologic findings.

The cases were histologically graded using the modified Bloom Richardson score system (MBRS).

## 3. Results

We have evaluated the surgical specimen of 40 cases (18 partial mastectomies and 22 mastectomies) following neoadjuvant chemotherapy. The technique used resulted in a total of 530 standard sections slides (average: 13 per case) and 52 large format sections (average: 1.3 per case).

Among these 40 cases there were 31 invasive ductal carcinomas, 8 invasive lobular carcinomas, and 1 mixed ductal and lobular carcinoma.

The cases were histologically graded using the modified Bloom Richardson score system (MBRS). The prechemo grades were low (5/9 MBRS) in two cases, intermediate (6-7/9) in eighteen cases, and high (8-9/9 MBRS) in twenty cases. The histopathologic grade of the tumors was an average of 7.4 prechemo and 4.8 post-chemotherapy.

The goal was to identify either residual viable cancer or the tumor bed in cases with complete response. Of the 40 cases, we have observed complete histopathologic response in 11 (27.5%) cases; near complete response was identified in 2 cases (defined as only rare clusters of residual invasive tumor cells involving an area equal or smaller than 1 mm). 27 (67.5%) cases had partial response; one of the cases with partial response had residual tumor cells only within lymphatic vessels without residual “infiltrating carcinoma”; another case had only residual disease in the axillary lymph nodes.

In these 40 cases, the average pretreatment tumor size was 3.5 cm by imaging studies. Inflammatory carcinoma and four quadrant disease were arbitrarily given a 10 cm measurement for purposes of pretreatment size estimation.

There was no significant difference between standard and large format slides in the identification of the tumor bed or the residual tumor, however in the large format is easier to see the spatial relationship and easier to be confident that a tissue edge in fact represents the margin and not an artifact created by the sectioning of the tissue.

Using standard sections the average post-treatment size was 1.8 cm; using large format, the average post-treatment size was 1.6 cm with a range of 0 to 10 cm for both. The post-treatment size reflects the overall area with tumor, either made up by scattered foci or by a single nodule of residual disease. This is easily measured in the large format slides by simply using a ruler and measuring 2 dimensions; the larger of the two is recorded as the final size of the tumor. For standard sections it is a combination of either measuring residual tumor when there are nodules smaller than 15 mm that can be measured on one slide or by adding the number of sequential slices with tumor multiplied by the thickness of the slices. An example would be a case where tumor is found in 3 slices and each slice measures 0.5 cm in thickness; this results in a residual tumor size of 1.5 cm.

**Figure 3:**
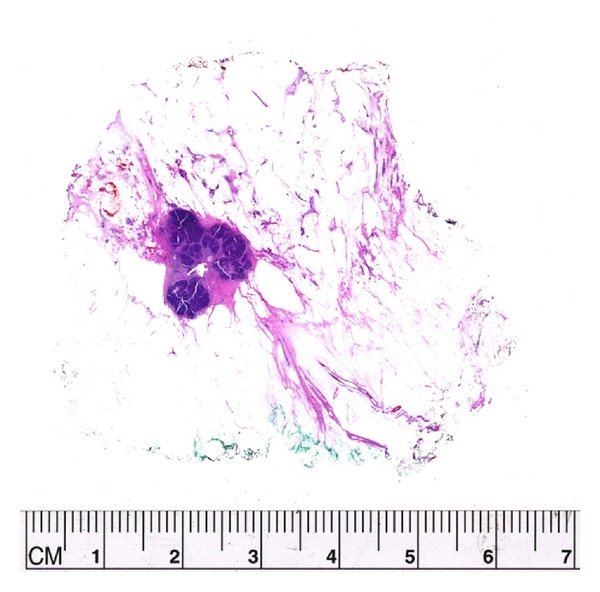
An example of “concentric regression” of tumor after neoadjuvant chemotherapy. She started with a 3 cm high grade (9/9 MBRS) invasive ductal carcinoma. At the end of treatment the tumor measured 1.4 cm.

**Figure 4:**
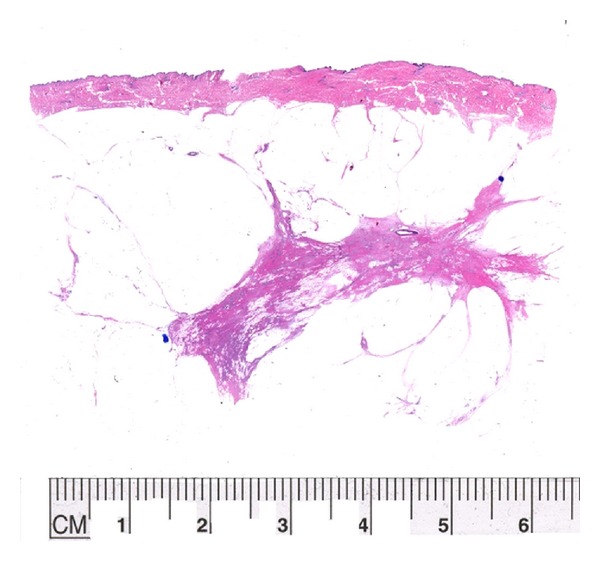
An example of “scatter regression” of tumor after neoadjuvant chemotherapy. She started with a 5 cm intermediate grade (6/9 MBRS) invasive ductal carcinoma. At the end of treatment the “tumor bed” with scattered foci of viable tumor cells involved an area of 4.7 cm represented by the irregular scar (density).

**Figure 5:**
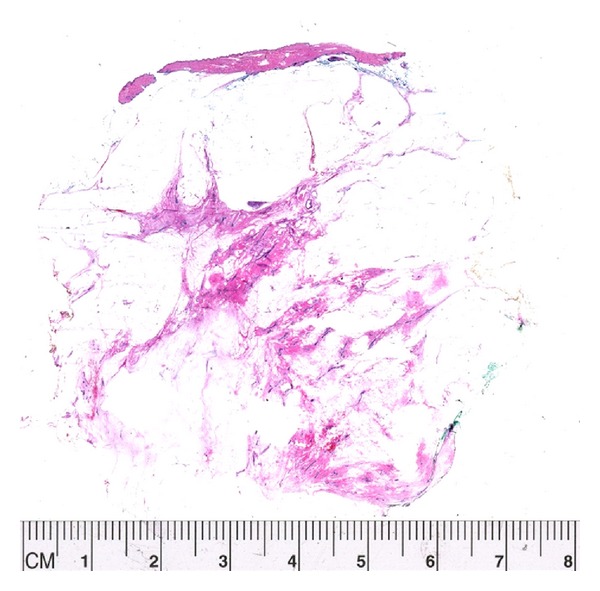
In this case, the patient had pathologic complete response (pCR). The entire specimen was examined microscopically. Slices one and three were cut in the perpendicular plane and slice 2 submitted for large format. A 100% of this 7.5 cm lumpectomy was examined microscopically with 12 standard sections and one large section.

**Figure 6:**
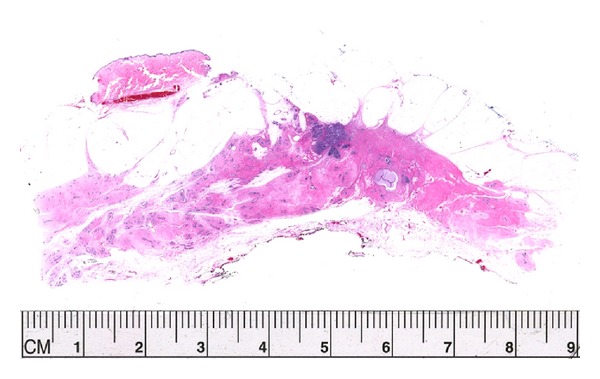
Example of “concentric regression” of tumor after neoadjuvant chemotherapy. She started with a 1.5 cm high grade (8/9 MBRS) invasive ductal carcinoma. After neoadjuvant chemotherapy she has a 0.6 cm focus of residual tumor.

**Figure 7:**
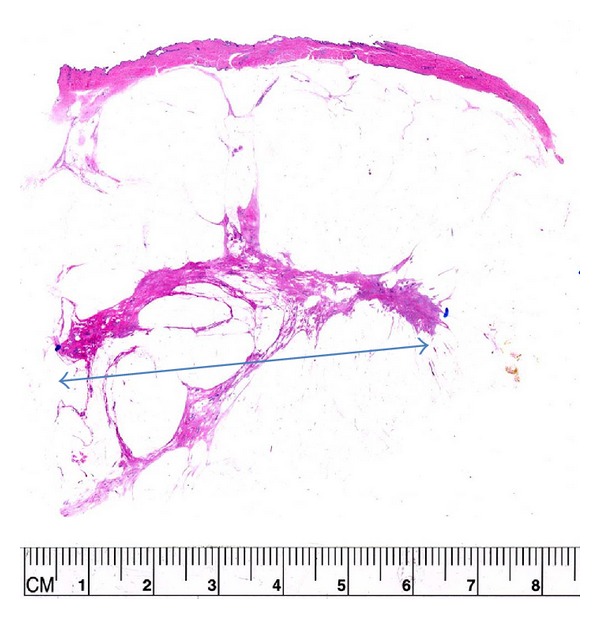
In this case, there is an area of 6 cm of residual invasive and in situ carcinoma. This would be difficult to measure in standard sections because of the difficulty in the orientation of the cut pieces.

Tumor regression has been described as “scatter” or “concentric” in type. Scatter cases are characterized by residual tumor cells, either singly or in clusters, identified within an area of the breast similar to the original tumor size. In Figure 3, an example of concentric regression, the tumor is composed of a dense 1.4 cm nodule of residual viable tumor. An example of the scatter pattern is seen in Figure 4 where the original tumor size was 5 cm and after treatment the residual scattered viable cells were present involving an area of 4.7 cm. These measurements are very difficult to obtain using standard sections. Figure 5 had originally a 2.5 cm tumor; after neoadjuvant therapy, the patient had complete imaging and clinical response. A large partial mastectomy with skin was performed and histopathologic examination showed complete response with proper identification of the tumor bed. Figures 6 and 7 demonstrate how simple it is to measure residual disease using the large format histopathology. In Figure 6 the patient had a 6 mm nodule of residual viable tumor. Likewise in Figure 7, the tumor size can be determined by using the caliper on the large section. Trying to measure the residual tumoral area by standard sections would be quite difficult because of the elongated nature of this lesion.

## 4. Discussion

The use of neoadjuvant chemotherapy has increased and is no longer limited to patients with locally advanced breast cancers; it is being used in patients who have relatively small tumors. The role of the pathologist is to assess the impact of chemotherapy on the primary breast cancer and/or its metastases to the axillary lymph nodes. The pathologist has to identify the location where the regressed tumor used to be (tumor bed) and identify the presence or absence of residual disease. This is accomplished by a close working relationship with the radiologist who usually inserts a metallic marker (fiducial marker) in the area of the tumor before the initiation of chemotherapy. Radiologic-pathologic correlation is critical and provides the most accurate results in the evaluation of cases after neoadjuvant chemotherapy [15]. After the patient has been treated, the tumor may be extremely difficult to see by the radiologist and no longer palpable by the clinician; therefore the surgeon has to rely on the radiologist to localize the “tumor bed” by placing a metal wire in the location of the fiducial marker. This way the surgeon knows with relative accuracy the area that needs to be removed. The volume of tissue that needs to be removed will depend on whether the tumor was a unifocal/multifocal or diffuse lesion. In cases of complete response the surgeon will be guided by localizing wires placed preoperatively by the radiologist. It will be the pathologist's responsibility to determine if the tumor bed has in fact been removed and whether the margins of resection are clear. Marchio and Sappino [16] reported that the use of large format histopathology was valuable in cases of neoadjuvant chemotherapy, particularly in the evaluation of the residual tumor burden and the status of the margins of resection. The margins are negative by definition in cases with complete pathologic response; however in cases where there is incomplete response, the disease may be microscopic and scattered over an area similar in size to the original area occupied by the intact tumor. It is in these cases when using the large section helps.

The comprehensive sampling of the circumferential margins performed in our cases is not the standard across the United States. Most laboratories submit random standard sections instead of the entire tissue slice. In a report by Tucker [17], he estimates that the average pathology practice examines 16% of the margins. Based on this incomplete information clinicians are making decisions every day regarding reexcisions and radiotherapy use.

Our collection of cases is not consecutive. The specific workup of the cases is quite unique with having the ability to compare the same area within a single slide (large format histopathology) versus 8–10 separate slides (standard histopathology). This type of comparison has not been done as far as we know.

Our cost estimation showed that the preparation of the 13 standard slides per case cost approximately $130.00 ($10.00 per slide) and for the 1.3 large format slides per case cost approximately $104.00 ($80.00 per slide); if we were to submit more of the tissue for large format and only the top and bottom margins for standard sections we would end up with small but real cost saving. For example, in a partial mastectomy with 4 tissue slices such as that depicted in Figure 2, we could submit 3 standard sections from the top and bottom slices (×6 slides = $60.00) and the two center pieces for large format slides (×2 slides = ($160.00) for a total of $220.00 per case.

## 5. Conclusion

We found that the combination of large format histopathology and standard sections provides accurate information in the identification of residual disease and margins width is easy to measure. For both, mastectomies and partial resections, we found no significant difference between large format and standard sections in the margin width or the size of the residual tumor or in the identification of the tumor bed in cases with complete histopathologic response. This is only true because of the extensive sampling utilized in these cases by standard sections and the fact that the large format slides are the mirror images of the standard sections. We recommend extensive sampling, either by large format or standard sections to accurately report the size of the residual tumor and the margin measurements.

One major advantage of the large format slides is the fact that we do not have to reassemble the “puzzle” using the standard sections. Finally, our cost analysis suggests that using primarily large format for our cases results in a slight cost savings ($208.00 versus $234.00) when compared with standard sections.

The correlation with imaging studies will be published in a separate paper but there is no doubt that it is much easier when large format histopathology is used.
